# Understanding Infection: A Primer on Animal Models of Periprosthetic Joint Infection

**DOI:** 10.1155/2013/925906

**Published:** 2013-09-22

**Authors:** Alexandra I. Stavrakis, Jared A. Niska, Amanda H. Loftin, Fabrizio Billi, Nicholas M. Bernthal

**Affiliations:** Orthopaedic Hospital Research Center, Orthopaedic Hospital Department of Orthopaedic Surgery, David Geffen School of Medicine at University of California, Center for Health Sciences (CHS), Rm 76-143, Los Angeles, CA 90095, USA

## Abstract

Periprosthetic joint infections are devastating complications for patients and for our health system. With growing demand for arthroplasty, the incidence of these infections is projected to increase exponentially. This paper is a review of existing animal models to study periprosthetic infection aimed at providing scientists with a succinct presentation of strengths and weaknesses of available in vivo systems. These systems represent the tools available to investigate novel antimicrobial therapies and reduce the clinical and economic impact of implant infections.

## 1. Introduction

Periprosthetic joint infection is a devastating complication of total joint arthroplasty. Despite advances in perioperative antibiotics and aseptic surgical technique, periprosthetic joint infection is currently the most common indication for revision total knee arthroplasty and the third most common indication for revision total hip arthroplasty in the United States [1]. Postarthroplasty infections occur in approximately 1% of primary arthroplasties and 3%–5% of revisions [2–6] and the incidence of infections continues to rise with the increasing demand for arthroplasty surgery. The annual number of total knee arthroplasties performed in the United States is estimated to reach 3.48 million by 2030, while the number of total hip arthroplasties is projected to increase to 572,000. With this projection of roughly 4 million arthroplasty surgeries performed each year by 2030, the annual incidence of arthroplasty infection is projected to rise from its 2005 estimate of 17,000 to a projected 266,000 by 2030 [3, 7, 8].

Arthroplasty infections are clinically devastating, often leading to multiple operations, prolonged hospitalization, and worse clinical outcomes. Severe infections often lead to amputation and can even result in death [9, 10]. These infections also pose a significant economic burden through direct medical costs and lost wages and productivity [7]. Medical costs alone average $144,514 per patient (compared with $30,173 for an uncomplicated arthroplasty) [9], which corresponds to a projected annual national healthcare burden of $8.63 billion by 2015 [8].

When arthroplasty implants become infected, they are exceedingly difficult to treat, especially when such an infection presents in a chronic setting. The bacteria produce a biofilm, a polysaccharide layer that blocks the penetration of antibiotics and cells of the host immune system [2, 11, 12]. The majority of such infections, approximately 70%, are caused by staphylococcal species. Over the past decade, hospitals have seen an increase in arthroplasty infection by antibiotic-resistant strains, the most common being methicillin-resistant *Staphylococcus aureus* [13–15].

Two fundamental criteria are used to classify periprosthetic joint infections: mechanism of infection and the timing of diagnosis. The mechanism of infection can be either direct seeding of the implant at the time of surgery or hematogenous spread of infection from elsewhere in the body. Surgeons attempt to prevent direct inoculation through sterile technique, perioperative antibiotics, and limiting duration of the operation. Once an implant is in place, there is concern for hematogenous spread any time the patient has an infection or transient bacteremia. Hematogenous spread of bacteria can be minimized by aggressively treating infections elsewhere in the body as well as prescribing prophylactic antibiotics for small procedures that lead to postoperative bacteremia (i.e., dental procedures). 

In terms of timing, infections that are identified in the first 4 weeks after surgery or within 4 weeks of another identifiable source of seeding (i.e., dental work or another surgical procedure) are categorized as “acute” infections. Infections greater than 4 weeks after index surgery and with no identifiable precipitating event are classified as “chronic” infections. This distinction is admittedly opaque but is based on our concept of a biofilm “establishing itself” on the implant over some period of time after seeding.

Current treatment protocols are driven by this timing-based classification. Acute infections are most often treated with irrigation and debridement, polyethylene liner exchange, and retention of the metallic components. Conceptually (although with little scientific validation), acute infections can be treated with irrigation and debridement because a mature biofilm has not yet been established. Good results have been reported with this technique [16]. Despite prompt management, irrigation and debridement of acute arthroplasty infections can result in recurrent infection [17]. In one study of acutely infected total knee arthroplasties treated with debridement, component retention, and intravenous antibiotics, only 35% of patients successfully retained their components at a four-year follow-up period. When a subset analysis was performed, only 8% of patients who were infected with *S. aureus* in comparison to 56% with *S. epidermidis* or streptococcal species were successfully treated via this method [18]. In addition to the virulence of the bacteria, other important prognostic factors also need to be considered prior to attempting component retention, such as the immune status and past medical history of the patient.

The current standard of care for treatment of chronic infections involves a multiple stage process beginning with surgical removal of all prosthetic components, debridement of the surrounding tissue, and placement of an antibiotic impregnated cement spacer. Patients are then placed on a 6-week course of intravenous antibiotics tailored to susceptibilities of the bacteria cultured from the surgery. Once this infection clears (as supported by a benign appearing wound, normal C-reactive protein, normal erythrocyte sedimentation rate, and negative joint aspiration cultures), a second-stage revision arthroplasty may be attempted [3]. In a prospective series, Mortazavi et al. reported that at an average of 3.4-year follow-up, 28% of patients who had a two-stage revision arthroplasty for an infected total knee arthroplasty required reoperation for infection [19]. In severe or persistent infections, long-term suppressive antibiotic therapy, arthrodesis, or even amputation is sometimes necessary [20, 21]. 

## 2. A History of Animal Models

There are a lot of interest and research on the prevention and treatment of implant infections since it is the single most common cause of arthroplasty failure. As in many areas of medicine, animal models have been used to better understand the pathophysiology of post-arthroplasty infection. Animal models are also an essential intermediary between *in vitro* laboratory work and clinical trials. 

The first joint infection animal model was established in rabbits and was published in the Journal of Bone and Joint Surgery in 1975 [22]. In this model, infection was tested in both a native knee and a knee with metal “implants” present. The “implant” arm of the study was made up of mice who had sterile stainless steel particles (<3 *μ*m in diameter) suspended in normal saline injected into the knee via the suprapatellar pouch. Infection was then produced via the inoculation of serial tenfold dilutions of a culture of either *Staphylococcus aureus* or Micrococcus species into the suprapatellar pouch. On postprocedure day 6 and at weekly intervals thereafter, cultures of the joint fluid were obtained via placement of a needle into the suprapatellar pouch, irrigation of the joint with 1.5 mL sterile saline, followed by aspiration and culture of the aspirate. In this study, stainless steel particles in the knee did not appear to increase susceptibility to infection from injected micrococci but did make established micrococcal infections more persistent [22]. However, because the metal was suspended in normal saline rather than implanted into the bone, there was question as to how appropriately this modeled the arthroplasty situation from both a bone-implant interface perspective as well as an opportunity for bacterial adherence. 

The first canine model was described by Petty et al. in 1985 [23]. Using a sterile technique, an incision was made over the tip of the greater trochanter and the bone was exposed subperiosteally. A hand drill and bone awl were used to penetrate the cortex and a 5 mm drill bit was used to ream the medullary cavity of the femur. The canal was then inoculated with the desired bacterial suspension (*Staphylococcus epidermidis*, *Staphylococcus aureus*, or *Escherichia coli*) and a 4 by 6 cm cylinder was introduced into the canal (stainless steel alloy, cobalt-chromium alloy, high-density polyethylene, or polymethylmethacrylate). The wound was then closed using a Dexon suture. At postoperative day 15, all the animals were euthanized and tissue was retrieved and cultured. The effects of the different implant materials on the susceptibility to infection were then compared. This model was later used to compare the effect of intraoperative irrigation and postoperative antibiotic treatment on infection rate [24]. One significant advantage of this animal model, in comparison to the rabbit model described previously, is that the metal implant used in this model (4 by 6 cm cylinder placed into the proximal femur) more closely represents an arthroplasty, in comparison to the stainless steel particles injected into the knee joint space in the prior model. Weaknesses of the model include the single, static data time point, postoperative day 15, and as a questionable surgical representation. Inoculating the bone and then placing an implant is perhaps a better model of introducing an implant into an existing osteomyelitis. The site of bacterial seeding is intraosseous, rather than intra-articular. 

This concern of intra-articular bacterial seeding was addressed by a novel arthroplasty infection rabbit model published by Craig et al. in 2005 [25]. A stainless-steel screw with an ultrahigh molecular weight polyethylene washer was cemented using polymethyl methacrylate in a defect created in an intra-articular, nonarticulating portion of the lateral femoral condyle of each knee. This was followed by inoculation of various concentrations of methicillin resistant staphylococcus aureus (MRSA). The animals were euthanized at postoperative day 7, at which time joint aspirate, tissues, and biomaterial samples were cultured. This model was also used to compare the infection rate of various biomaterials (i.e., polymethyl methacrylate (PMMA), bone cement, ultra high molecular weight polyethylene (UHMWPE), and stainless steel) [25]. One advantage of this model was that bacteria were introduced directly into the knee joint following wound closure whereas older models inserted bacteria directly into the femoral canal prior to implant placement or immersed biomaterials in a bacterial suspension prior to intra-articular placement. This method of inoculation more closely modeled an arthroplasty infection. A second advantage of this model was that it included the major biomaterials used in total knee arthroplasty, with use of PMMA, UHMWPE, and metal. 

**Figure 1:**
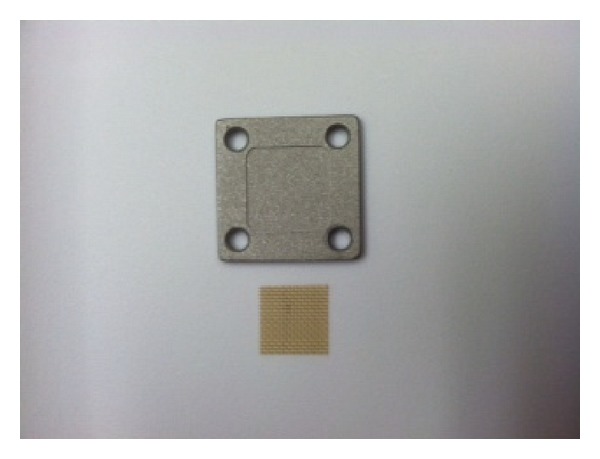
Photograph of a stainless steel plate and the PEEK membrane used for preimplantation formation of biofilm used in the Williams sheep model. *Courtesy of Dr. D. Williams*.

**Figure 2:**
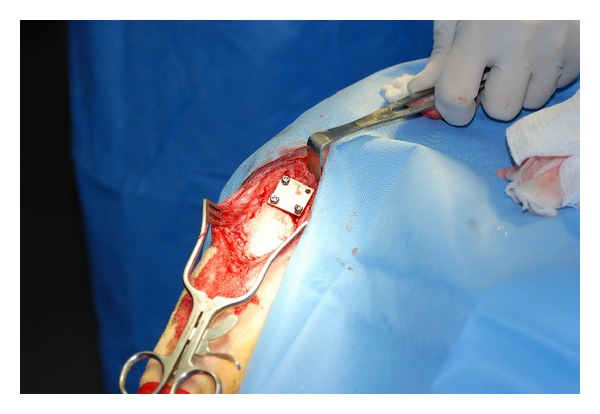
Intraoperative photographs of stainless steel plates placed in the proximal tibia used in the Williams et al. sheep model. *Courtesy of Dr. D. Williams*.

Another modification addressed by recent work in animal modeling has been based on the observation that a significant number of hardware infections may be the result of inoculation by mature bacterial biofilms rather than independent bacteria [26–32]. Williams et al. explored this observation and hypothesize that using a biofilm as initial inocula, rather than native bacteria, may provide more clinically relevant information for the prevention and treatment of hardware infections. In this model, a clinical isolate of methicillin-resistant *S. aureus* was used and was grown on the surface of membranes composed of polyetheretherketone (PEEK) for 48 hours. The biofilms were then isolated and used as an inoculum in a Gustilo type IIIB open tibia fracture model in sheep. An anterior midline sagittal incision was made from the tibial tuberosity extending distally along the anterior aspect of the tibia. In order to mimic a type IIIB Gustilo open fracture with significant periosteal stripping, bone exposure, and massive contamination, a section of periosteum was removed from the proximal anteromedial aspect of the tibia. A construct consisting of a stainless steel plate with a membrane containing the biofilm was placed against the tibial surface with the biofilm membrane between the plate and cortical bone. Each plate was then secured with cortical bone screws and the incision was finally closed with suture (Figures 1 and 2). Postoperatively, the wound was observed for signs of infection such as erythema, warmth, and dehiscence. At 12 weeks postoperatively, the sheep were euthanized and several samples were cultured, including the incision site, the subcutaneous tissue, the plate, bone, and the biofilm membrane. Radiographic and histological analyses were also performed from these samples. All sheep in the group inoculated with the biofilm membrane showed signs of infection, specifically osteomyelitis, at the 12-week postoperative period in comparison with no infection in any of the sheep treated without the biofilm. These findings strongly support the hypothesis that biofilms can cause infection. Although this model examines infection in an open fracture model rather than an arthroplasty model, it provides a key modification in animal modeling of arthroplasty with this concept of biofilm inoculation as the inciting event [33].

**Figure 3:**
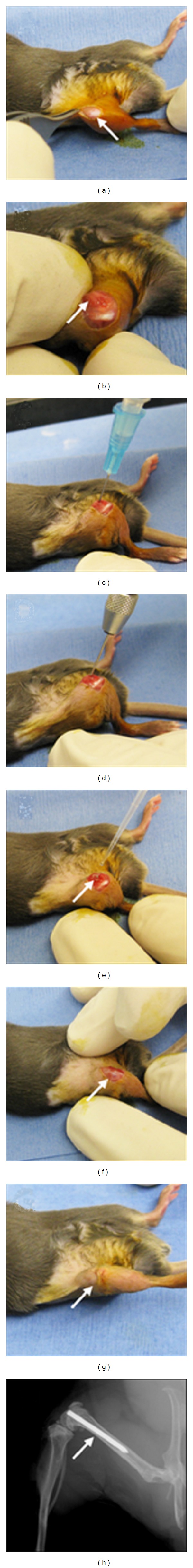
((a)–(g)) Bernthal et al. surgical approach in a representative mouse. (h) A radiograph demonstrating placement of the implant in the femoral canal with the cut end extending into the knee joint [3].

**Figure 4:**
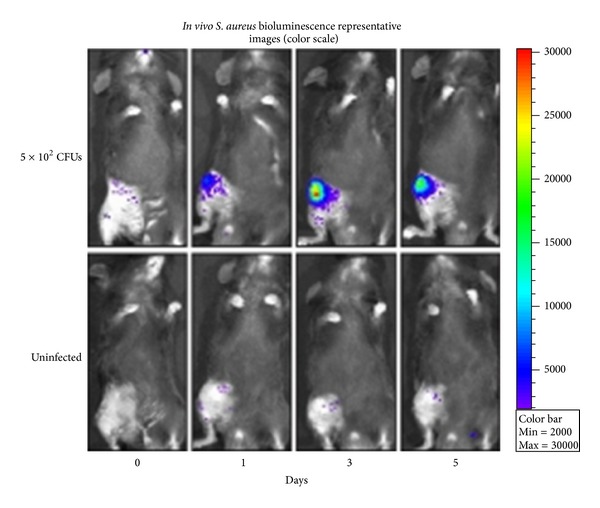
Representative *in vivo* bioluminescent images [3].

Such studies using histology and culture data provide extremely useful preclinical information; however, these studies are costly and labor intensive and require the use of a significant number of animals, as euthanasia is required to determine the bacterial burden at each time point postoperatively. In 2010, Bernthal et al. published a novel mouse model for post-arthroplasty infection that abdicated this need, using *in vivo* imaging of bioluminescent bacteria to replace histologic assessment [3]. Following a medial parapatellar approach to the knee, a metal pin was placed retrograde, from the knee joint into the femoral canal with 1 mm of the pin remaining protruding into the joint space. A bioluminescent strain of bacteria was then used to inoculate the intra-articular portion of the metal pin in the joint space (Figure 3). Postoperatively, the Xenogen *in vivo* imaging system was used to monitor the infection by quantifying bacterial burden in real-time (Figure 4). The *in vivo* bioluminescent signals were confirmed to accurately represent the bacterial burden *in vivo* by performing traditional bacterial counts on the last day of imaging. The initial model was created with use of a stainless steel Kirschner wire and a bioluminescent strain of *S. aureus*. This model was then applied to test a variety of biomaterials and various bacterial strains. This model has unique elements that may complement or provide an alternative to the use of other previous animal models. One unique characteristic of this model is that it uses advanced techniques of *in vivo* imaging, which provides longitudinal, real-time quantification of bacterial burden. Thus, an infection in a certain animal can be followed over several days or even weeks (may simulate an acute, subacute, or chronic post-arthroplasty infection). This bypasses the need to euthanize a large number of animals at subsequent time points to quantify bacterial burden. Genetically modified mouse lines are readily available, which can also be helpful in studying post-arthopalsty infections. For example, the use of various immunologic knockout mice or mice with fluorescent immune cells may aid in understanding the complex immune response against such infections [34]. 

## 3. Conclusions

Researchers have come a long way since the initial animal model of arthroplasty infection in 1975. The development of novel scientific techniques, from biofilm harvesting to *in vivo* imaging has provided opportunities to improve animal models to a more accurate and humane depiction of the human condition. And yet, each iteration along the way has made an important contribution. The ideal model offers the anatomic similarities to human joints that a large animal model offers, the immunogenic modulation available in a mouse model, the longitudinal data collection that bioluminescence offers, and potentially, the use of biofilm inoculation that was recently described [31]. While all of these assets may not be available in a single model, one could devise a combination of existing models that utilizes the strengths of small animal modeling as an initial high-throughput screen and large animal modeling as a preclinical test. Additionally, future models would ideally be able to test a representative panel of bacteria, more accurately representing the clinical scenarios that patients and clinicians face. 

As the prevalence of periprosthetic infection continues to rise alongside the increasing demand for arthroplasty, there is a great need to identify both preventative and therapeutic options. Such treatment strategies will continue to depend on animal models as an intermediary between bench concepts and clinical care. Thus, developing an appropriate, efficient and accurate animal model or series thereof is of the utmost importance.
